# ﻿Two new species of genus *Leucoagaricus* (Agaricaceae, Agaricales) from Pakistan

**DOI:** 10.3897/mycokeys.96.101745

**Published:** 2023-04-10

**Authors:** Shazia Ashraf, Arooj Naseer, Muhammad Usman, Abdul Nasir Khalid

**Affiliations:** 1 Fungal Biology and Systematics Research Laboratory, Institute of Botany, University of the Punjab, Quaid-e-Azam Campus 54590, Lahore, Pakistan University of the Punjab Lahore Pakistan

**Keywords:** Bayesian analysis, Islamabad, Margalla, systematics

## Abstract

The genus of basidiomycetous fungi, *Leucoagaricus*, occurs worldwide, from subtropical to boreal latitudes. Several collections of *Leucoagaricus* were made during mycological field trips conducted in different forests of Margalla, Pakistan. An integrative framework combining morphological and phylogenetic data was employed for their study. As a result, the two species *La.margallensis* and *La.glareicolor* are here described as new to science. Detailed macro- and micro-morphological descriptions, and a molecular phylogenetic reconstruction based on nrITS and LSU sequence data are provided and used to discriminate the new species from morphologically and phylogenetically close taxa. Whereas, our phylogenetic tree inference gave unequivocal support for the inclusion of these two species within the section Leucoagaricus.

## ﻿Introduction

The genus *Leucoagaricus* Locq. ex Singer, is a relatively well known mushroom-forming genus of basidiomycetous fungi, characterized by the small to medium-sized, thin or fleshy basidiomata; pileus surfaces ranges from radially fibrillose, floccose, squamulose to fibrillose-scaly or rarely granulose; entire or very short striated margins; and central, equal to bulbous stipe that have membranous, sometimes movable annuli; thin-walled and smooth basidiospores generally lack well-defined germ pores; and the pileipellis is either a trichoderm or a cutis of repent and radially arranged hyphae lacking sphaerocysts (Singer 1986; Vellinga 2001).

Taxonomic studies on *Leucoagaricus* throughout the whole of Pakistan are, in fact, scant. Only 12 species of *Leucoagaricus* have been reported from Pakistan so far (Ahmad et al. 1997; Ge et al. 2015; Qasim et al. 2015; Hussain et al. 2018; Usman and Khalid 2018; Ullah et al. 2019; Asif et al. 2021). All in all, further research on the diversity of this genus in the whole region is required. The aim of the present work is to provide new insights about the diversity of *Leucoagaricus* species from Pakistan.

## ﻿Materials and methods

### ﻿Morphological and anatomical studies

Basidiomata were collected following Lodge et al. (2014) and photographed in their natural habitats using a Nikon D70S camera. Morphological features were recorded from fresh specimens. Colors were designated with reference to mColorMeter application (Yanmei He, Mac App Store). Collections of the newly described species were deposited in the Herbarium of the Department of Botany, University of the Punjab, Lahore, Pakistan (acronym LAH). Microscopic characters are based on freehand sections from fresh and dried specimens mounted in 5% (w/v) aqueous Potassium Hydroxide (KOH) solution and examined using a Meiji Techno MX4300H compound microscope. A total of 30 basidiospores, basidia, cystidia and hyphae from pilei were measured from each collection. For basidiospores, the abbreviation “*n/m/p*” indicates *n* basidiospores measured from *m* fruit bodies of *p* collections. Dimensions for basidiospores are given using length × width (L × W), and extreme values are given in parentheses. The range contains a minimum of 90% of the values. Measurements include the arithmetic mean of spore length and width.

### ﻿Laboratory procedures, sequence alignment and phylogenetic analyses

Genomic DNA was extracted from portions of lamellae following a modified CTAB extraction method (Bruns 1995). ITS and LSU regions of nuclear rDNA were amplified using the pairs of primers ITS1F-ITS4B and LR0R-LR5 (Vilgalys and Hester 1990; White et al. 1990; Gardes and Bruns 1993). Polymerase chain reactions (PCR) were performed in a total volume of 25 μL and consisted of an initial 4 minutes denaturation step at 94 °C, 40 cycles of 1 minute at 94 °C, 1 min at 55 °C, 1 min at 72 °C, and a final extension step of 10 minutes at 72 °C. Visualization of PCR products on a 1.5% agarose electrophoretic gel was done staining with SYBR Green. Successful amplicons were purified by enzymatic purification using Exonuclease I and Shrimp Alkaline Phosphatase enzymes (Werle et al. 1994). Bidirectional sequencing of purified products was done by Macrogen (Republic of Korea). Chromatograms were checked and assembled using SeqmanII v.5.07 (Dnastar Inc.). Once sequences were assembled and edited they were deposited in GenBank (http://www.ncbi.nlm.nih.gov).

The online tool BLAST and the databases GenBank (http://www.ncbi.nlm.nih.gov/) was used to check for possible PCR-product contamination and to identify and retrieve available, highly similar *Leucoagaricus* nrITS and LSU sequences to the newly produced sequences. A comprehensive representation of currently available sequences, in NCBI database with similarity up to 92% identity and 95% query cover for ITS Phylogenetic tree and 95% identity and 98% query cover for LSU Phylogenetic tree, were used for the phylogenetic analyses and all the sequence of section Leucoagaricus from recent publications were also included. The final dataset consists of 54 sequences as ingroup and one sequence of *Cystolepiotaseminuda* (Lasch) Bon (AY176350 for ITS and AY176351 for LSU) from the Netherlands was used as outgroup. The dataset for the phylogenetic tree was made by MUSCLE alignment in SEA VIEW software version 5.0.5 (Gouy et al. 2010). The final Maximum Likelihood phylogram was made in RAxML-HPC2 using XSEDE tool (8.2.10) with 1000 bootstrap values. We used jModelTest 2.1.6 (Darriba et al. 2012) to verify the best nucleotide substitution model, using the Akaike information criterion. The Maximum Likelihood analyses were performed using RAxML v.8 (Stamatakis 2014), with the GTRGAMMA model and 1000 replicates. Phylogenetic analyses were based on maximum likelihood (ML) and Bayesian (B/MCMC) approaches, performed on the Cipres Science Gateway webserver (https://www.phylo.org/). The ML analysis was performed using RAxML v.8 (Stamatakis 2014), with the GTRGAMMA model and 1000 bootstrap replicates. Branches with bootstrap values ≥ 80% for ML were considered to be supported.

For the tree reconstruction based on Bayesian inference, the program MrBayes 3.2.7 (Ronquist et al. 2012) was used with two parallel Markov chain Monte Carlo (MCMC) chains with 10 million generations, saving every 1000^th^ tree. The first 25% of the sampled trees was discarded as burn-in and 50% percent majority rule tree was generated along with posterior probabilities (PPs) of ≥0.80 by FigTree v. 1.4.2 (Rambaut 2012). Newly generated sequences were deposited in GenBank.

## ﻿Results

### ﻿Phylogenetic analyses (Figs 1, 2)

Sequences of the nr DNA ITS region basidiomata MH63 (LAH37453), MH111 (LAH37454), MH65 (LAH37575) and MH169 (LAH37456) were obtained with both primers and the final sequences consisted of 780, 770, 753, and 656 base pairs, respectively and 1090, 936, 977 and 953 base pairs, respectively for LSU. These four samples belong to two different taxa described here under the name of *Leucoagaricusmargallensis* and *Leucoagaricusglareicolor*. For ITS, the aligned final dataset comprised 719 characters including gaps; out of these, 422 characters were conserved, 286 were variable, 199 were parsimony informative and 87 were singletons. For LSU, the aligned final dataset comprised 947 characters including gaps; out of these, 763 characters were conserved, 178 were variable, 131 were parsimony informative and 47 were singletons. Our taxa are separating from their closest species with strong bootstrap value of 100 for ML and 1 PP for BI and there was no conflict in both analyses in the position of our taxa.

**Figure 1.**
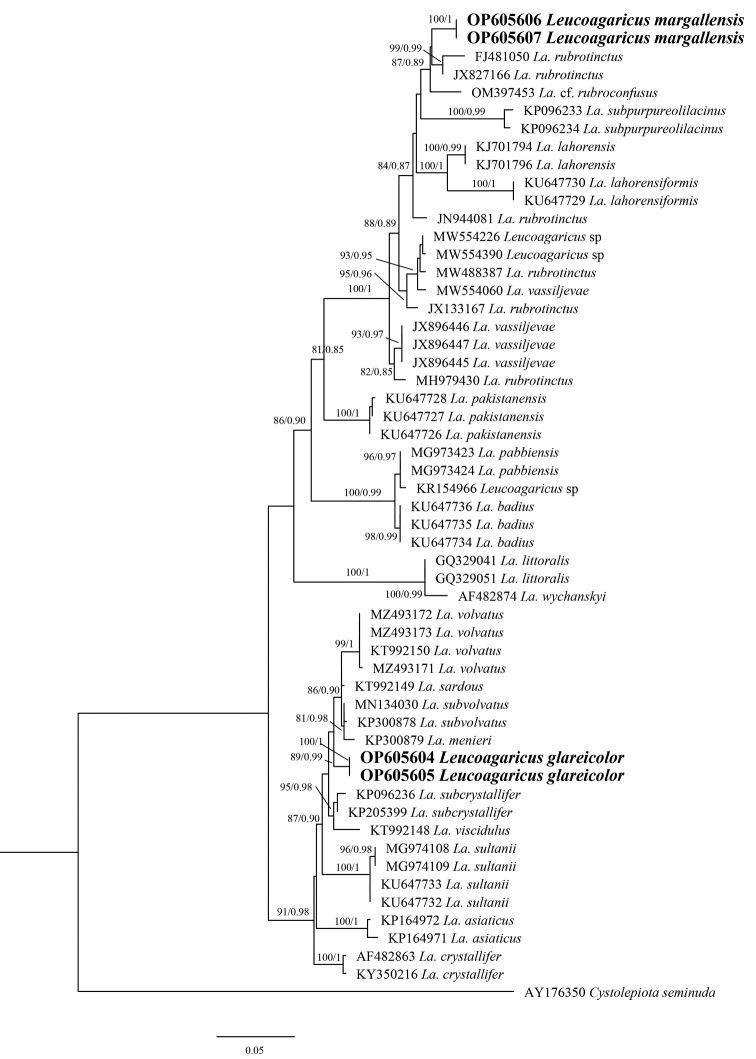
Molecular phylogenetic analyses by maximum likelihood (ML) and Bayesian Inference (BI) method based on ITS sequences. Bootstrap and Posterior probability values are shown at the branches as ML/BI and novel sequences generated during this study are in bold.

**Figure 2.**
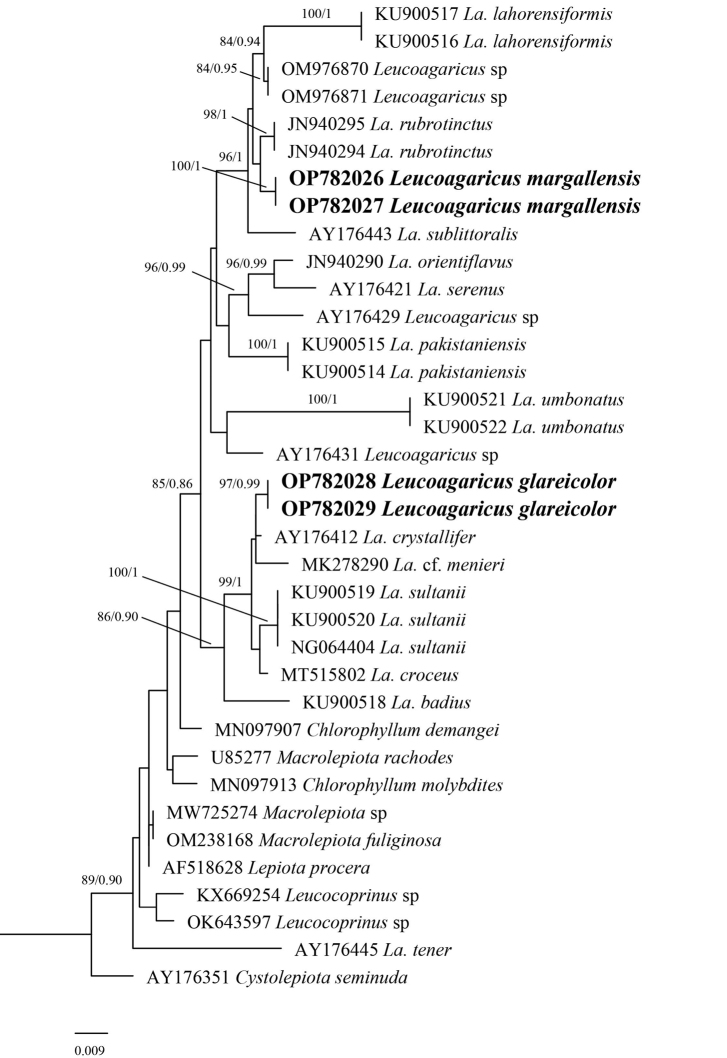
Molecular phylogenetic analyses by maximum likelihood (ML) and Bayesian Inference (BI) method based on LSU sequences. Bootstrap and Posterior probability values are shown at the branches as ML/BI and novel sequences generated during this study are in bold.

## ﻿Taxonomy

### ﻿ Leucoagaricus margallensis

Taxon classificationFungiAgaricalesAgaricaceae

Ashraf, S., Naseer, A. & Khalid, A.N. sp. nov.

67196D18-B574-54AB-BEAA-1362442FF60D

846300

Figs 3 , 4

#### Etymology.

The specific epithet *margallensis* (Latin) refers to type locality Margalla.

#### Diagnosis.

*Leucoagaricusmargallensis* can be distinguished by small, umbonate pileus with minute, fragile annulus, bulbous stipe, smaller basidiospores (6.27 × 4.67 µm) and cheilocystidia without crystals on its apex.

#### Holotype.

Pakistan, Islamabad, Margalla Hills National park, trail 5, 33°45'01.1"N, 73°05'14.5"E, 1303 m.a.s.l., July 27, 2018, Shazia Ashraf & Arooj Naseer, MH-63, Holotype (LAH 37543), GenBank: OP605606 (ITS), OP782026 (LSU).

#### Description.

***Basidiomata*** medium-sized, shiny, smooth, moderately fleshy, solitary. ***Pileus*** 1.5–2.5 cm in diameter, hemispherical to parabolic when young, expanding convex to plano convex, plane at maturity, umbonate, radially fibrillose to rugulose, whole pileus orange (0.5YR 3.2/8.7) when immature, then yellowish orange (9.4YR 6.1/7.2) on maturity, margin incurved in mature and striated, context white, smooth, thick at center. ***Lamellae*** free to thin, milky white (2.2GY 6.8/0.9), spacing fine and close, 1–1.5 mm wide, edges entire, lamellae regular, 3-4 tiers. ***Lamellulae*** irregular, alternating with lamella, in 2-3 tiers. ***Stipe*** 4.5–7 × 0.5–1 cm, cylindrical, central, thin, white (2.2GY 6.8/0.9), smooth, shiny, even, clavate. Annulus simple, white, located in upper half of stipe, membranous upturned. Flavor and odor not distinctive.

**Figure 3.**
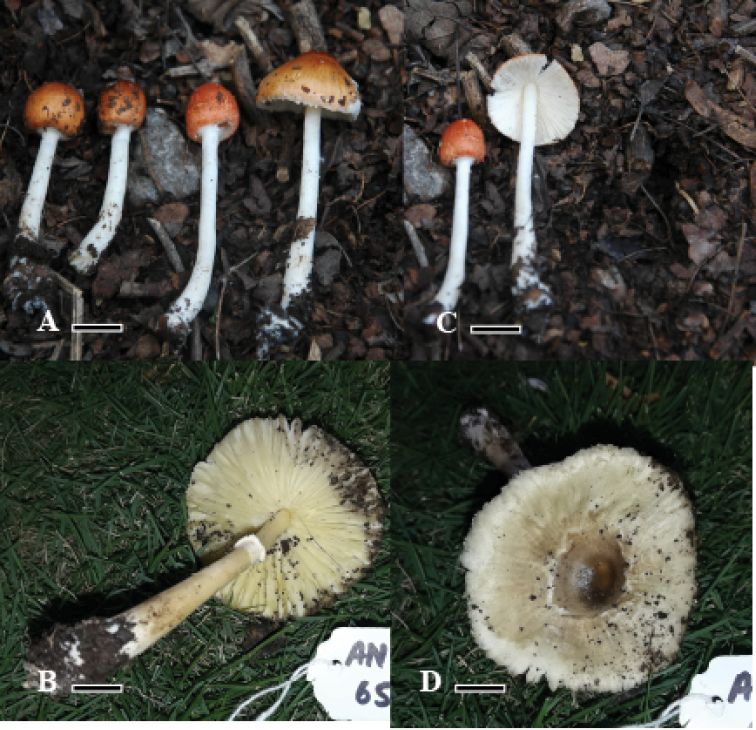
Morphological description of *Leucoagaricus* spp. from Pakistan **A, C***Leucoagaricusmargallensis***B, D***Leucoagaricusglareicolor*.

***Basidiospores*** [90/5/3] (6–) 6.2 – 8.0 (–8.2) × (3.43–) 4.11 – 4.7(–4.9) µm, 6.1–8.1 × 3.7–4.8 µm, av. Q = 1.58–1.42, Qav = 1.49, hyaline to light yellowish in 5% KOH, oval to ellipsoid at face view, lacrymoid to amygdaliform in side-view, guttulate, smooth, dextrinoid, thin-walled and apiculate. ***Basidia*** (12.06–) 13.08 – 23.5 (–24.03) × (6.86–) 7.7 – 8.72 (–9.72) µm, 12.9–18.5 × 5.8–7.7 µm, av. L =19.29 µm, av. W = 7.54 µm, narrowly clavate, hyaline in 5% KOH, smooth, with 2–4 prominent sterigmata, oil droplets present,no clamp at base. ***Cheilocystidia*** (15.12–) 16.12 – 24.9 (–25.98) × (6.88–) 7.8 – 8.4 (–9.43) µm, 15–26 × 6.8–9.4 µm, hyaline in 5% KOH, thick-walled, broadly clavate and smooth, without internal content and clamp. Pleurocystidia absent. Pileipellis an intricate trichoderm, made up of 5.6–7.7 µm, av w=5.99 µm, wide, septate, interwoven, thin-walled hyphae, hyaline in 5% KOH, clamp connections absent.

**Figure 4.**
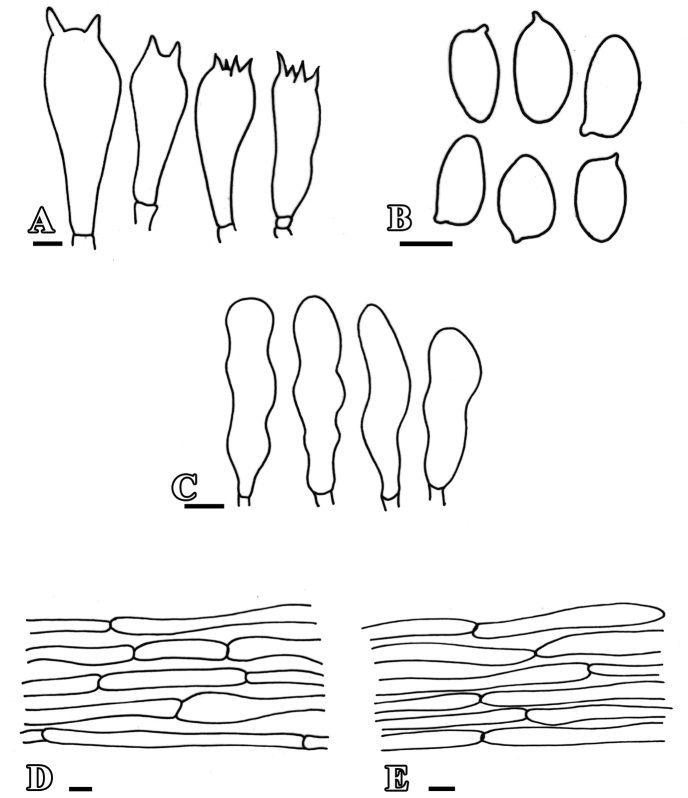
Anatomical features of *Leucoagaricusmargallensis***A** basidia **B** basidiospores **C** cheilocystidia **D** pileipellis **E** stipitipellis. Scale bars: 10 µm (**A**); 5 µm (**B–E**).

Stipitipellis as compactly arranged cutis, made up of septate, cylindrical hyphae, 3.4–4.9 µm in diam., thin-walled, light yellowish in 2% KOH, clamp connections absent.

#### Additional specimen.

Pakistan, Islamabad, Margalla Hills National park, trail 5, 33°45'01.1"N, 73°05'14.5"E, 1303 m a.s.l., July 22, 2021, Shazia Ashraf, MH-111(LAH 37454), GenBank for ITS (OP605607) LSU (OP782027).

#### Comments.

*Leucoagaricusmargallensis* has a combination of striking features like small, umbonate pileus with minute, fragile annulus and broader bulbous stipe. Anatomically, it has smaller basidiospores (6.27 × 4.67 µm), smaller cheliocystidia and absence of crystals at apex of cheliocystidia.

*Leucoagaricusmargallensis* resembles *La.rubrotinctus* including similar pileus size and shape, fibrillose surface, stipe color and shape. But *Leucoagaricusmargallensis* differs from *La.rubrotinctus* by minute fragile annulus while *La.rubrotinctus* has prominent white annulus. Anatomically, *Leucoagaricusmargallensis* has ellipsoid, smaller (6.27 × 4.67 µm) basidispores as compared to *La.rubrotinctus* larger and amygdaliform basidiospores. Furthermore, *Leucoagaricusmargallensis* has smaller cheliocystidia (15–26 × 6.8–9.4 µm) as compared to *La.rubrotinctus* (30–50 × 5–10 µm), narrow pilleipellis hyphae (5–10 µm) in *La.rubrotinctus*. The other closely related taxa in phylogenetic tree is *L.rubroconfusus* Migl. & Coccia, characterized by centrally depressed, larger (up to 7.5 cm) pileus with orange fibrillose squamules as compared to umbonate, smaller (2.5 cm) yellowish orange fibrils on white pileus of Pakistani taxon. Anatomically, both taxa lack crystals at apex of cheilocystidia. Our taxon *Leucoagaricusmargallensis* is differentiated from *L.rubroconfusus* due to smaller (6.27 × 4.67 µm) basidiospores as compared to basidiospores of *L.rubroconfusus* (5.5–8(9.5)) µm.

*Leucoagaricussubpurpureolilacinus* Z.W. Ge & Zhu L. Yang, from southwestern China differs in its broad, brown to dark ruby umbo, larger basidiospores and clavate cheilocystidia with gelatinized covering intermixed with crystals (Ge et al. 2015). Our taxon can also be differentiated from *La.purpureolilacinus*. *Leucoagaricuspurpureolilacinus* has a pinkish brown pileus with a dark purple brown disk, a stipe attenuating toward the base, amygdaliform spores and clavate cheilocystidia often with constrictions in the middle (Vellinga 2001).

### ﻿ Leucoagaricus glareicolor

Taxon classificationFungiAgaricalesAgaricaceae

Ashraf, S., Naseer, A. & Khalid, A.N. sp. nov.

C3B75EE3-5A39-5E48-9E87-AE8E8CEAE363

846302

Figs 3 , 4 , 5

#### Etymology.

The specific epithet *glareicolor* (Latin) refer to the brownish black color of umbo and fibrils on pileus.

#### Diagnosis.

Is distinguished due to brown to blackish brown, broadly umbonate, white pileus with blackish brown fibrillose to rugulose, smooth sub-bulbous stipe with annulus in upper 1/3 of stipe, smaller (5.65–7.70 × 3.44–5.63 µm) basidiospores and cheilocystidia without crystals at apex.

#### Holotype.

Pakistan, Islamabad, Margalla Hills National Park, Trail 5, 33°45'01.1"N, 73°05'14.5"E, 1303 m a.s.l., August 28, 2018, Shazia Ashraf & Arooj Naseer MH-169 (LAH37456) GenBank: OP605605 (ITS) and OP782028 (LSU)

#### Description.

***Basidiomata*** medium to large-sized, soft shiny, fragile solitary. ***Pileus*** 4-4.2 cm, plano-convex to plane at maturity, radially fibrillose to rugulose towards margins, broadly umbonate, umbo brown when young, becoming blackish brown with age, disc creamy white (1.7GY 6.4/1.6) with dark brown (3.8Y 3.5/3.2) to blackish brown (4.8Y 5.5/8.2) rugulose towards margins, shiny and sericeous, on maturity broadly striated and overextended blackish brown fibrils darker in center and lighter towards margin, margin incurved and appendiculate. ***Lamellae*** free to approximate, adnexed, distant, fragile, edges entire, yellowish creamy (1.7GY 6.4/1.6) to grayish brown (4.8Y 5.5/8.2). ***Lamellulae*** irregular, alternating with lamella, in 2-3 tiers. ***Stipe*** 6.2–6.7 cm in length, tapering upwards, 0.5 cm at apex, 1.7 cm at base, sub bulbous, yellowish creamy (1.7GY 6.4/1.6) central, cylindrical, smooth, shiny. ***Annulus*** present, superior, thin, made up of cottony scales, non-persistent in nature. Flavor and odor not distinctive. ***Basidiospores*** [60/3/2] (5.65–) 5.66 – 7.73 (–7.76) × (3.43–) 4.11 – 4.6 (–5.63) µm, 5.65–7.70 × 3.44–5.63 µm, Q = 1.23–1.70, Qav = 1.48, hyaline in 5% KOH, oval at face view, lacrymoid to amygdaliform in side-view, guttulate, smooth, dextrinoid, thin-walled and apiculate. ***Basidia*** (12.06–) 13.08 – 23.5 (–24.03) × (6.86–) 7.7 – 8.72 (–9.72) µm, 12.9–18.5 × 5.8–7.7 µm, narrowly clavate, hyaline in 5% KOH, smooth, with 2–4 prominent sterigmata, oil droplets present, no clamp at base. ***Cheilocystidia*** (15.12–) 16.12 – 24.9 (–25.98) × (6.88–) 7.8 – 8.4 (–9.43) µm, 15–26 × 6.8–9.4 µm, hyaline in 5% KOH, thin-walled, clavate to narrowly clavate, smooth, without internal content and clamp. ***Pleurocystidia*** absent. Many layers of isodiametric irregular epithelial cells av = 7.87 µm in diameter, at the base of basidia and cystidia. ***Pileipellis*** trichoderm, made up of septate, cylindrical hyphae 5.6–7.7µm in diam., thin-walled, light brown to reddish pigment in the center, light orange to yellowish pigment towards margin in 2% KOH, clamp connections absent. ***Stipitipellis*** cutis compactly arranged, made up of septate, cylindrical hyphae 3.4–7.1µm in diam., av. w = 5.86 µm thin-walled, hyaline in 2% KOH, clamp connections absent.

#### Additional material examined.

Pakistan, Islamabad, Margalla Hills National park, Trail 5, 33°45'01.1"N, 73°05'14.5"E, 1303 m.a.s.l., July 27, 2018 Shazia Ashraf & Arooj Naseer, MH-65 (LAH37555), GenBank for ITS OP605604 & OP782029 for LSU).

#### Comments.

*Leucoagaricusglareicolor* is characterized by broad umbo that is brown to blackish brown, fibrillose to rugulose white pileus. The stipe is smooth, sub-bulbous with annulus that is present in upper 1/3 of stipe. Anatomically, *Leucoagaricusglareicolor* has smaller (5.65–7.70 × 3.44–5.63 µm) basidiospores and cheilocystidia without crystals at apex.

*Leucoagaricusglareicolor* shows close relationship with *L.subvolvatus* and *L.menieri*. *Leucoagaricussubvolvatus* and *L.glareicolor* share many similarities like same pileus color, size and shape. However, *L.subvolvatus* has broad yellow umbo with yellow, fine fibrils while *L.glareicolor* has very prominent, blackish brown umbo with blackish brown fibrils. *Leucoagaricussubvolvatus* is characterized by stipe base with a marginal bulb. *Leucoagaricusglareicolor* has slightly bulbous stipe. Furthermore, *Leucoagaricussubvolvatus* has annulus at lower part of stipe while *Leucoagaricusglareicolor* has prominent annulus in upper 1/3 half of stipe. Furthermore, *Leucoagaricussubvolvatus* has cheilocystidia with crystals at apex whereas *Leucoagaricusglareicolor* cheilocystidia lacks crystals at apex (Candusso and Lanzoni 1990).

Compared to *Leucoagaricusmenieri*, characterized by light yellow, slightly umbonate pileus which is milky white while *Leucoagaricusglareicolor* has brownish black umbo, creamy white pileus with blackish brown fibrils. The stipe in the former is more bulbous (4–5 mm) as compared to subbulbous (0.5–1.7 cm) stipe in *Leucoagaricusglareicolor*. Anatomically *Leucoagaricusmenieri* is differentiated from *Leucoagaricusglareicolor* by longer basidiospores {(6.9–)7.4(–8.7)} and the presence of crystals at apex of cheilocystidia. Another closely related taxon is *Leucoagaricussardous* that is differentiated by our taxa by broader {5–6.8 (7.3)} basidiospores and larger (upto 75 µm) cheilocystidia. The next closely related taxon is *L.volvatus* that is characterized by the gelatinized, white pileus with olivaceous tinges and presence of crystals on cheilocystidia.

**Figure 5.**
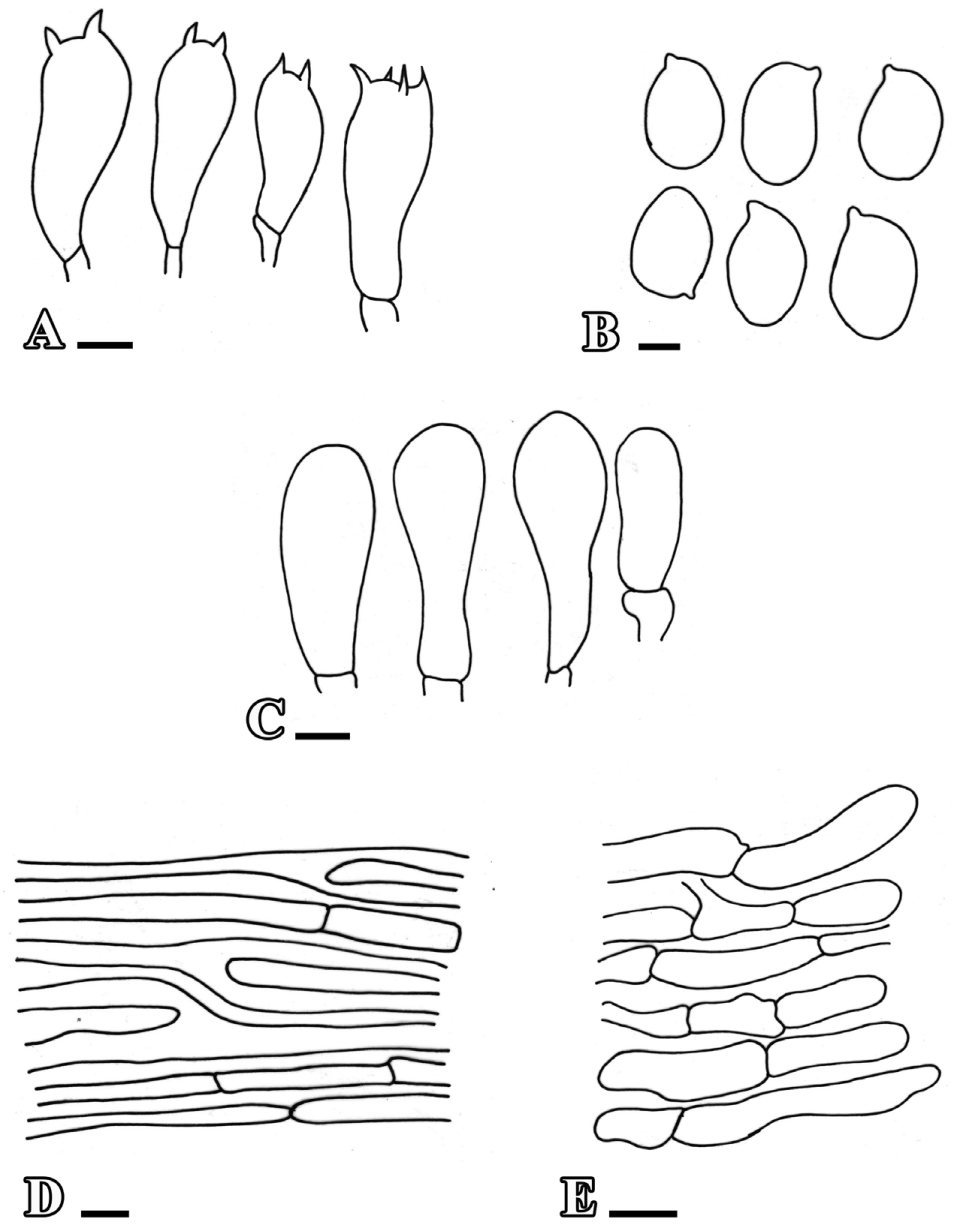
Anatomical features of *L.glareicolor***A** basidia **B** basidiospores **C** cheilocystidia **D** pileipellis **E** stipitipellis. Scale bars: 5 µm (**A–C**); 10 µm (**D, E**).

Molecular phylogenetic analyses based on ITS and LSU sequences also support *La.glareicolor* as a distinct species with strong bootstrap support.

## ﻿Discussion

In this paper, two new species of *Leucoagaricus* were studied morphologically and sequences of two DNA regions were analyzed for each species. With the new data provided in the present study, the number of *Leucoagaricus* species for all of Pakistan increases to fourteen. All in all, these data suggest that our knowledge of the diversity of *Leucoagaricus* in high mountain areas in Asia and the Margala forests of Pakistan is still in its infancy. These two new species provide evidence that further research is needed to collect and identify the fungal diversity of Asia.

## Supplementary Material

XML Treatment for Leucoagaricus margallensis

XML Treatment for Leucoagaricus glareicolor
